# Characterizing the epidemiology, virology, and clinical features of influenza in China’s first severe acute respiratory infection sentinel surveillance system, February 2011 – October 2013

**DOI:** 10.1186/s12879-015-0884-1

**Published:** 2015-03-22

**Authors:** Zhibin Peng, Luzhao Feng, Greene M Carolyn, Kaili Wang, Guozhong Zhu, Yequn Zhang, Jumei Hu, Yiwei Huang, Huiqiong Pan, Nongjian Guo, Chunyan Xing, Yanhui Chu, Zhaolong Cao, Deshan Yu, Linling Liu, Zeling Chen, Fang Zeng, Wen Xu, Xin Xiong, Xiuwei Cheng, Hua Guo, Wu Chen, Ling Li, Hui Jiang, Jiandong Zheng, Zhen Xu, Hongjie Yu

**Affiliations:** Division of Infectious Disease, Key Laboratory of Surveillance and Early-warning on Infectious Disease, Chinese Center for Disease Control and Prevention, Beijing, China; Centers for Disease Control and Prevention, Atlanta, Georgia; Heilongjiang Provincial Centers for Disease Control and Prevention, Haerbin, China; Heilongjiang Provincial Hospital, Haerbin, China; HuzhouCenter for Disease Control and Prevention, Huzhou, China; Huzhou First People’s Hospital, Huzhou, China; Hunan Provincial Centers for Disease Control and Prevention, Changsha, China; The First Hospital of Changsha, Changsha, China; Jinan Center Hospital, Jinan, China; XichengCenter for Disease Control and Prevention, Beijing, China; Peking University People’s Hospital, Beijing, China; Gansu Provincial Centers for Disease Control and Prevention, Lanzhou, China; The First Hospital of Lanzhou University, Lanzhou, China; Zhuhai Centers for Disease Control and Prevention, Zhuhai, China; Zhuhai People’s Hospital, Zhuhai, China; Yunnan Provincial Centers for Disease Control and Prevention, Kunming, China; The First Affiliated Hospital of Kunming Medical University, Kunming, China; Sichuan Provincial Centers for Disease Control and Prevention, Chengdu, China; The Third People’s Hospital of Chengdu, Chengdu, China; Fujian Provincial Centers for Disease Control and Prevention, Fuzhou, China; The First Affiliated Hospital of Fujian Medical University, Fuzhou, China

**Keywords:** Epidemiology, Virology, Clinical features, Influenza positive patients, China

## Abstract

**Background:**

After the 2009 influenza A(H1N1)pdm09 pandemic, China established its first severe acute respiratory infections (SARI) sentinel surveillance system.

**Methods:**

We analyzed data from SARI cases in 10 hospitals in 10 provinces in China from February 2011 to October 2013.

**Results:**

Among 5,644 SARI cases, 330 (6%) were influenza-positive. Among these, 62% were influenza A and 38% were influenza B. Compared with influenza-negative cases, influenza-positive SARI cases had a higher median age (20.0 years *vs.*11.0, p = 0.003) and were more likely to have at least one underlying chronic medical condition (age adjusted percent: 28% *vs.* 25%, p < 0.001). The types/subtypes of dominant strains identified by SARI surveillance was almost always among dominant strains identified by the influenza like illness (ILI) surveillance system and influenza activity in both systems peaked at the same time.

**Conclusions:**

Data from China’s first SARI sentinel surveillance system suggest that types/subtypes of circulating influenza strains and epidemic trends among SARI cases were similar to those among ILI cases.

## Background

**Figure 1:**
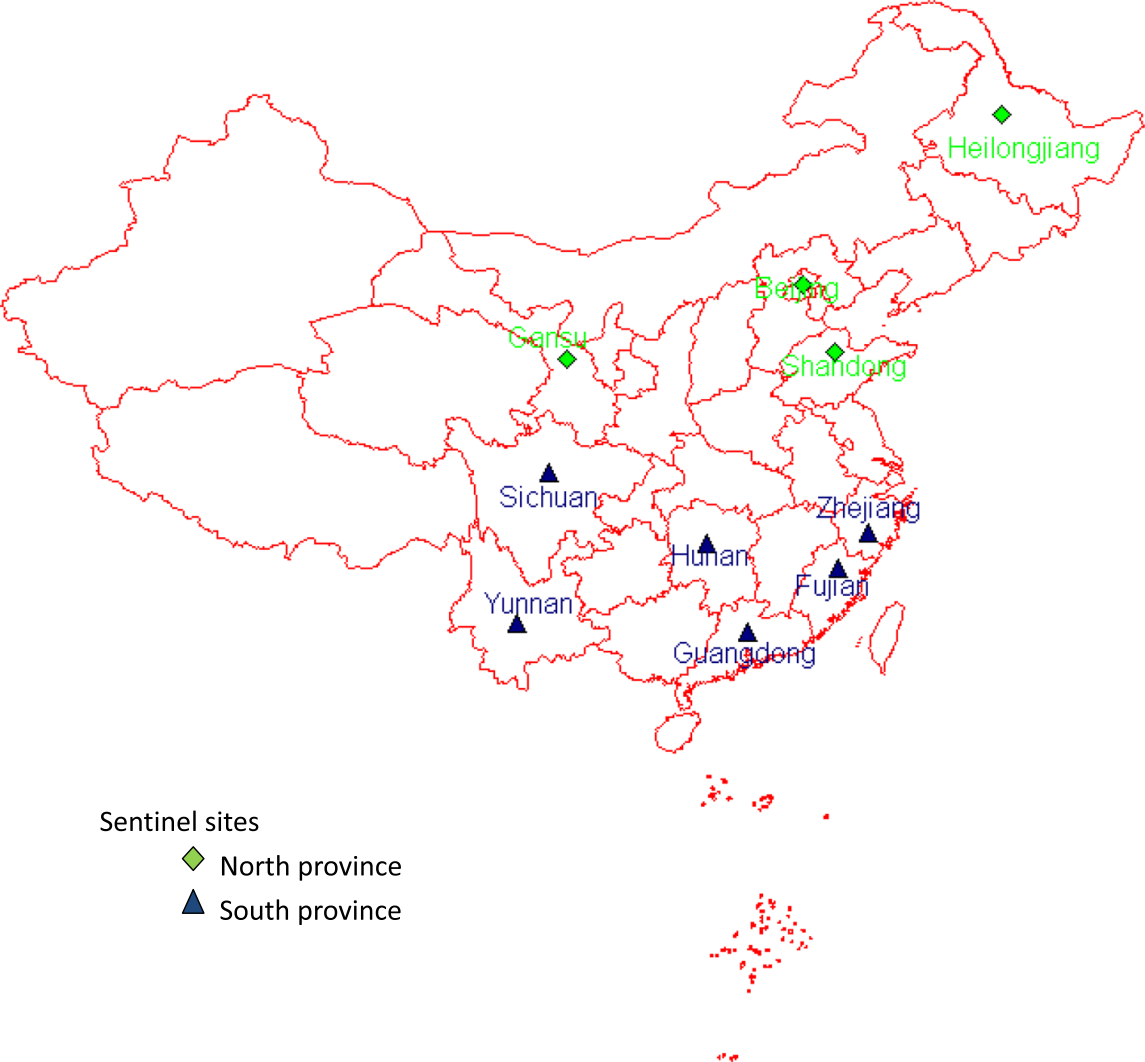
**Location of 10 sentinel sites for severe acute respiratory infection (SARI) surveillance in China.** The Qinling Mountain – Huaihe River serves as the dividing line between north and south China. This border is located at 32- 34°northern latitude. North China has a monsoon climate of medium latitudes, while south China has a subtropical monsoon climate.

In 2005, China established a national influenza-like illness (ILI) surveillance system to monitor influenza activity and to describe the epidemiology of influenza among outpatients and the virologic characteristics of circulating virus [1]. The A(H1N1)pdm09 influenza pandemic of 2009 underscored the importance of establishing systems to monitor severe disease. When the World Health Organization reissued its recommendation for Member States to develop surveillance systems for severe acute respiratory infection (SARI) among inpatients [2,3], China selected 10 sentinel hospitals in 10 provinces and their corresponding influenza laboratories from the national ILI surveillance network to initiate SARI surveillance among hospitalized patients [Figure 1]. These hospitals were selected based on regional representativeness and demonstrated capacity to conduct ILI surveillance. In February 2011, the Ministry of Health released a revised protocol to enhance surveillance of severe respiratory infections and more specifically, severe infections with laboratory-confirmed influenza [4].

We analyzed data from China’s first SARI surveillance system during its initial 33 months, from February 2011 to October 2013, to characterize the epidemiology and clinical characteristics of SARI and laboratory-confirmed influenza patients, and to describe the seasonal trends of influenza in China.

## Methods

### Setting

Sentinel surveillance was conducted at ten general hospitals located in the largest cities of ten provinces in China. In each hospital, surveillance was conducted within the pediatrics ward, the respiratory medicine ward and the intensive care unit. The number of beds in these wards combined was greater than 80 in each of the ten hospitals.

### Patient enrollment

All patients admitted to surveillance wards at the 10 hospitals were screened by nurses and physicians for SARI. A patient >5 years old was defined as having SARI if, upon or during admission, they presented with an acute onset of elevated temperature (axillary temperature ≥38°C) and cough or sore throat, AND tachypnea (respiratory rate ≥ 25/min) or dyspnea (difficulty breathing). A patient ≤5 years old was defined as having SARI if, upon or during admission, they presented with acute onset of cough or dyspnea, AND at least one of the following six signs or symptoms: a) tachypnea (respiratory rate >60/min for ages <2 months, respiratory rate >50/min for ages 2 to <12 months, and respiratory rate >40/min for ages 1 to ≤5 years); b) inability to drink or breastfeed; c) vomiting; d) convulsions; e) lethargy or unconsciousness; f) chest in-drawing OR stridor in a calm child [5]. Per China’s Ministry of Health national influenza surveillance guidelines implemented in October 2009, collection and analysis of data for this project were considered official public health surveillance activities and institutional review board approval was not required [6].

### Patient data collection

Upon enrollment, physicians registered the SARI case-patient’s name, gender, and date of illness onset. During the hospital admission, physicians completed a standardized case report form (CRF) that recorded demographic information, chronic medical conditions, signs and symptoms of current illness, and laboratory and radiographic data. At hospital discharge, physicians updated the form with information on clinical course during hospitalization, including treatment received, complications and outcomes.

### Specimen collection and testing

Nurses collected nasopharyngeal (NP) swabs from SARI case-patients within 24 hours of enrollment following standardized procedures. Swabs were immediately placed in viral transport medium (VTM) and stored at 4°C at the local hospital. These specimens were transferred to the closest influenza network laboratory (provincial or prefecture Center for Disease Control and Prevention (CDC) laboratories) within 48 hours of collection. The local influenza network laboratories stored the specimens in VTM at −70°C until they conducted testing by real-time reverse transcription PCR (rRT-PCR) following the standard protocol [7,8].

### Data analysis

Based on influenza activity, climate and geography, we divided China into two distinct regions, north and south, for the analysis and interpretation of influenza surveillance data. The Qinling Mountain – Huaihe River serves as the dividing line between north and south China [9-12]. In our sentinel surveillance system, 4 hospitals are located in north China, while 6 are located in south China [Figure 1].

Hospital infection control and local CDC staff entered epidemiologic, clinical, and laboratory data into an electronic database that was transmitted weekly to the national China CDC. We defined a patient with laboratory-confirmed influenza as any SARI case-patient with an NP swab that tested positive for influenza virus by rRT-PCR. We analyzed data that were collected from February 1, 2011- October 27, 2013 with SPSS (v13.0, SPSS, Chicago, IL, USA). Descriptive statistics included frequency analysis for categorical variables such as gender, age group, underlying chronic medical conditions, clinical characteristics and outcomes. We calculated medians and interquartile ranges for continuous variables such as age and length of clinical course. Differences in demographic and clinical characteristics and outcomes were assessed among SARI patients with and without laboratory-confirmed influenza using the chi-squared test or the Fisher’s exact test for nominal variables. We calculated age-adjusted mortality rates using standard techniques [13], with the 2010 China population census serving as the reference population [14]. We used the Wilcoxon rank sum test for ordinal variables. Differences were considered statistically significant at *P* < 0.05.

### ILI surveillance in China

Ten hospitals included in the sentinel SARI surveillance system were also ILI sentinel hospitals within the national influenza surveillance system. We compared influenza activity among SARI and ILI patients using surveillance data from the outpatient departments of the ten hospitals in the SARI surveillance system from February 2011 – October 2013.

The Chinese national influenza-like illness (ILI) surveillance system currently includes 409 network laboratories and 554 sentinel hospitals. Surveillance is conducted in sentinel hospital emergency rooms and internal medicine and pediatric outpatient departments. Each surveillance department registers the number of ILI patients seen each day to calculate the proportion of all medical visits that are due to ILI [8]. NP swabs are collected from a sample of ILI patients, and are sent to the nearest network laboratory for influenza testing with rRT-PCR and additional virologic analyses.

## Results

From February 1, 2011 – October 27, 2013, 155,639 patients were hospitalized in the surveillance wards of the ten sentinel hospitals. Among these, 5,644 (4%) met the case definition for SARI and NP swab specimens were collected for all 5,644. CRFs were completed for 5,268 (93%). Among all SARI cases, 330 (6%) tested positive for influenza by rRT-PCR, and among these, CRFs were completed for 299 (91%).

### Characteristics of SARI patients

Of the 5,268 SARI patients with completed CRFs, 794 (14.2%) were children <1 year old, 1386 (26.3%) were children 1 to <5 years old, and 1050 (19.9%) were adults ≥65 years old [Table 1]. SARI patients had a median age of 12.0 years (interquartile range [IQR], 2.0 - 58.0 years), and 3091 (59%) were male.

**Table 1:** **Characteristics of hospitalized severe acute respiratory infection (SARI) patients and laboratory-confirmed influenza patients in 10 sentinel surveillance hospitals in 10 provinces, China, February 1, 2011 to October 27, 2013**

**Characteristics**	**All SARI patients (%) [n = 5,268]** ^**a**^	**SARI patients with confirmed influenza (%) [n = 299]** ^**a**^	**SARI patients without confirmed influenza (%) [n = 4,678]** ^**a**^	***p*** **value**
Male sex	3,091 (58.7)	193 (64.5)	2,725 (58.3)	0.202
Age, median (interquartile range [IQR], years)	12.0 (2.0-58.0)	20.0 (3.0-67.0)	11.0 (2.0-56.0)	**0.003** *
**Age group**				
<6 months	357(6.8)	11 (3.7)	335 (7.2)	**<0.001** *
6 - 11 months	392 (7.4)	15 (5.0)	362 (7.7)	
12 - 23 months	458 (8.7)	24 (8.0)	411 (8.8)	
2 - 4 years	928 (17.6)	51 (17.1)	836 (17.9)	
5 - 9 years	412 (7.8)	37 (12.4)	351 (7.5)	
10 - 14 years	137 (2.6)	7 (2.3)	125 (2.7)	
15 - 49 years	976 (18.5)	38 (12.7)	886 (18.9)	
50 - 64 years	554 (10.5)	35 (11.7)	476 (10.2)	
≥65 years	1050 (19.9)	81 (27.1)	896 (19.2)	
Underlying chronic medical conditions				
At least one^b^	1407 (26.7)	111 (37.1)	1296 (27.7)	**<0.001** *
Cardiovascular disease	792 (15.0)	64 (21.5)	728 (15.6)	**<0.001** *
Chronic obstructive pulmonary disease^c^	198 (3.8)	23 (7.7)	175 (3.7)	0.064
Diabetes mellitus	244 (4.6)	22 (7.4)	222 (4.7)	0.215
Chronic bronchitis	205 (3.9)	22 (7.4)	183 (3.9)	**0.049** *
Cancer/Tumor	138 (2.6)	13 (4.3)	125 (2.7)	**0.043** *
Asthma	85 (1.6)	2 (0.7)	83 (1.8)	**0.040** *
Vaccinated with seasonal influenza vaccine during past year	47 (1.1)	6 (2.7)	41 (1.1)	**0.041** *
Clinical history and physical examination				
Fever	4440 (84.3)	284 (95.0)	4156 (88.8)	**<0.001** *
Highest temperature after onset, median (interquartile range [IQR], °C)	39.0 (38.5-39.5)	39.0 (38.6-39.6)	39.0 (38.5-39.5)	0.072
Abnormal breath sounds on auscultation	2950 (60.0)	196 (65.6)	2754 (58.9)	0.071
Cough	4235 (80.4)	256 (85.6)	3979 (85.1)	0.592
Sore throat	1661 (31.5)	103 (34.4)	1558 (33.3)	**0.028** *
Difficulty breathing	1449 (27.5)	112 (37.5)	1387 (29.6)	**0.012** *
Clinical pneumonia				
Abnormal chest X-ray performance	2868 (54.4)	176 (58.9)	2692 (57.5)	0.992
Diagnosis of clinical pneumonia	1189 (22.6)	66 (22.1)	1123 (24.0)	0.925
Clinical course, median (IQR), days				
From illness onset to hospital admission	3 (1–6)	2.5 (1–5)	3 (1–6)	0.802
Length of stay in hospital	8 (6–12.5)	9 (6.75-14)	8 (5–12)	0.593
Admission to ICU^d^	61 (1.2)	5 (1.7)	56 (1.2)	0.840
Death^e^	98 (1.9)	12 (4.0)	86 (1.8)	**0.013** *

^a^Data are presented as no. (%) of patients unless otherwise indicated. Denominators for testing are indicated. Percentages may not total 100 because of rounding. As a proportion of SARI case patients did not have influenza testing results, the sum of all patients with and without confirmed influenza is not equal to the total number of SARI patients.

^b^At least one underlying medical condition defined as: admission diagnosis of any of the following: chronic obstructive pulmonary disease, asthma, cardiovascular disease, diabetes mellitus, chronic bronchitis, or cancer/tumor.

^c^Chronic obstructive pulmonary disease defined as a lung disease characterized by chronic obstruction of lung airflow that interferes with normal breathing and is not fully reversible.

^d^ICU denotes intensive care unit.

^e^During hospitalization.

*The symbol bold data means *p*<0.05.

Just under 85% of SARI case-patients reported a temperature of ≥38°C after illness onset, with a median peak temperature of 39.0°C (IQR, 38.5 - 39.5°C). After fever, the most common clinical symptom or sign was cough (80%). Among SARI patients, 1,189 (23%) were clinically diagnosed with pneumonia during their hospital admission, and 2,868 (54%) had at least one abnormal finding on chest X-ray. The median duration of hospitalization was 8 days (IQR, 6–12.5), and 61 (1.2%) patients were admitted to an intensive care unit. Ninety-eight (1.9%) patients died during their hospitalization. Among SARI case-patients, 1,407 (27%) had at least one chronic medical condition, such as cardiovascular disease, chronic obstructive pulmonary disease, diabetes mellitus, chronic bronchitis, cancer/tumor, and asthma.

### Characteristics of patients with laboratory-confirmed influenza

**Figure 2:**
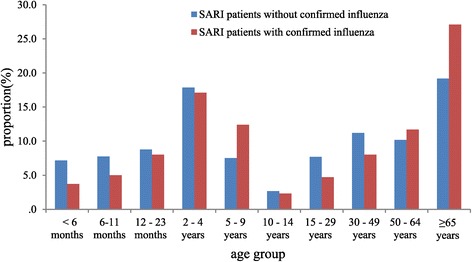
**Proportion of severe acute respiratory infection (SARI) patients with and without confirmed influenza by age group, China, Febuary 1, 2011 to October 27, 2013.**

Of 299 SARI patients with laboratory-confirmed influenza, 101 (34%) were <5 years old, 145 (48%) were <15 years old, and 81 (27%) were ≥65 years old [Table 1]. The distribution of cases by age category was different among SARI cases with and without confirmed influenza [Figure 2]. The proportion of SARI cases with confirmed influenza was higher among those aged > = 65 years (27% *vs*. 19%, p = 0.001) and those 5–9 years old (12.4% *vs* 7.5%, p = 0.002) and lower among those <1 year old (8.7% *vs.* 14.9, p = 0.003) and those aged 15–49 years (12.7% *vs.* 18.9%, p = 0.007). Influenza types/subtypes detected included A(H1N1)pdm09 influenza (pH1N1) (100, 33%), A(H3N2) (80, 27%), A (subtype not identified) (4, 1%), and influenza B (115, 38%). The SARI patients with confirmed influenza had a significantly higher median age than those without confirmed influenza (20.0 years *vs.* 11.0, p = 0.003), and more often had at least one chronic medical condition (37% *vs.* 28%, p < 0.001; age adjusted percentage 28% *vs.* 25%, p < 0.001) including cardiovascular disease (22% *vs.* 16%, p < 0.001), chronic bronchitis (7%*vs. *4%, p < 0.05), and cancer/tumor (4% *vs.* 3%, p < 0.05) [Table 1]. Although asthma was uncommon in both groups, it was less common among SARI patients with confirmed influenza (1% *vs.* 2%, p < 0.05). Compared with SARI patients without confirmed influenza, those with confirmed influenza more frequently had fever (95% *vs. *89%, p < 0.001), sore throat (34% *vs. *33%, p < 0.05) and difficulty breathing (38% *vs. *30%, p < 0.05). SARI patients with confirmed influenza were no more likely to have clinical pneumonia than SARI patients without confirmed influenza (22.1% *vs.* 24.0%, p = 0.925). Mortality was higher among influenza positive SARI patients than influenza negative patients (4.0% *vs.* 1.8%, p = 0.013), but there was no significant difference after age adjustment (2.7% *vs.* 1.5%, p = 0.137). Receipt of seasonal influenza vaccination in the prior year was low among both SARI patients with and without confirmed influenza (2.7% *vs.* 1.1%, p = 0.041).

Of 1,260 SARI patients with clinical pneumonia, 1,189 (94%) had an NP swab specimen tested, and 66 (6%) tested positive for influenza virus including pH1N1 (21, 32%), A(H3N2) (11, 17%), and influenza B (28, 42%).

### Temporal trends

#### North 4 sentinel hospitals

**Figure 3:**
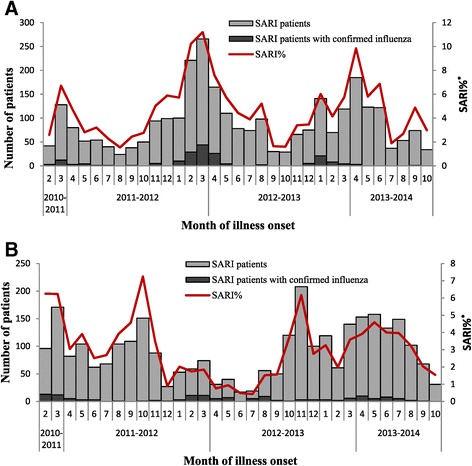
**Hospitalized severe acute respiratory infection (SARI) patients and patients with confirmed influenza virus infection by month of illness onset, China, Febuary 1, 2011 to October 27, 2013.** Panel **A**. Four Northern Hospitals: SARI patients (N=2,780) and patients with confirmed influenza virus infection (n=187). Panel **B**. Six Southern Hospitals: SARI patients (N=2,858) and patients with confirmed influenza virus infection (n=143). *SARI% = proportion of severe acute respiratory infection (SARI) patients among all hospitalized patients in the surveillance wards.

**Figure 4:**
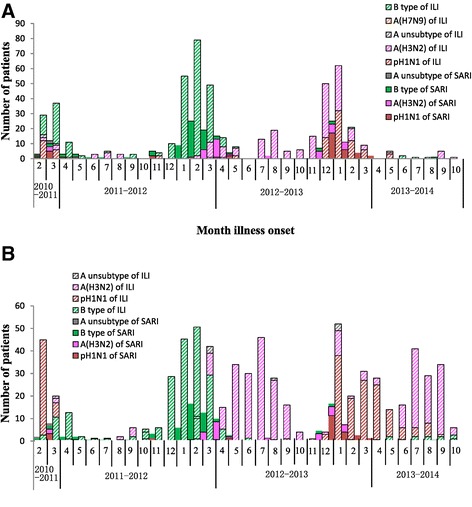
Number of influenza positive by type/subtype and by month of illness onset among hospitalized severe acute respiratory infection (SARI) patients and influenza like illness (ILI) outpatients in ten sentinel hospitals, China, Febuary 1, 2011 to October 27, 2013*. Panel **A**. Four Northern Hospitals. Panel **B**. Six Southern Hospitals. *The bars with diagonal slashes indicate isolates from ILI patients, while the solid bars indicate isolates from SARI patients.

In the 4 sentinel hospitals in the north, there was a winter-spring seasonal peak (December to May each year) in SARI patients during the 33 month surveillance period [Figure 3, *Panel* A]. Similarly, there was an increase in influenza activity during the winter (December to February) and spring months (March to May) [Figure 3, *Panel A*]. During the study period, the predominant types/subtypes of influenza virus in the north based on isolates from SARI patients were different for each seasonal peak of influenza activity; pH1N1 predominated during the 2011 spring peak (accounting for 33% of isolates collected March-May), B during the 2011–2012 winter peak (accounting for 87% of isolates collected December-February), A(H3N2) during the 2012 spring months (accounting for 50% of isolates collected March-May), and pH1N1 during the 2012–2013 winter months and 2013 spring months (accounting for 57% of isolates collected December-February and 100% of isolates collected March-May 2013) [Figure 4, *Panel A*].

### South 6 sentinel hospitals

In the south, data from the 33 month surveillance period revealed a winter-seasonal peak in SARI patients [Figure 3, *Panel B*]. There was also an increase in influenza activity during the winter (December to March) and spring months (March to May). In addition, there was an increase in influenza activity during the summer months (July to August) [Figure 3, *Panel B*]. In contrast to the north, in the 2011–2012 and 2012–2013 influenza seasons in the south, influenza activity peaked during months when the numbers of hospitalized SARI cases were not at their highest. However, in the 2013–2014 season, influenza activity and SARI cases peaked at the same time. The predominant types/subtypes of influenza virus in the south based on isolates from SARI patients were pH1N1 during 2011 spring months (accounting for 35% of isolates collected March-May), B during 2011–2012 winter months (accounting for 71% of isolates collected December-February), A(H3N2) during 2012 spring months (accounting for 64% of isolates collected March-May), A(H3N2) and B during 2012 summer months (accounting for 63% and 37% of isolates collected July-August), and pH1N1 during the 2012–2013 winter months, 2013 spring months and 2013 summer months (accounting for 53% of isolates collected December-February; 83% of isolates collected March-May and 63% of isolates collected July-August) [Figure 4, *Panel B*]. Similar to the north, the predominant types/subtypes of circulating influenza virus in the south for both the winter and spring months of every influenza season were the same.

### Comparison with influenza like illness (ILI) surveillance

**Figure 5:**
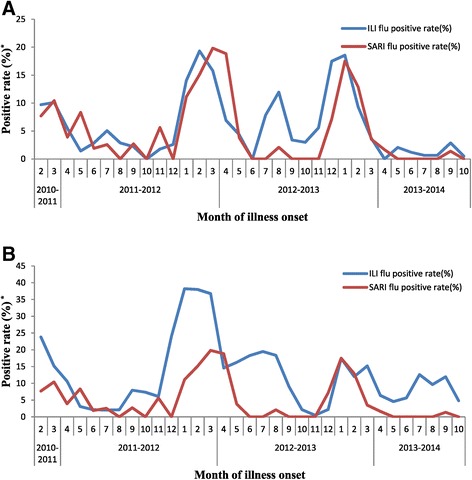
**Percent of influenza positive by month of illness onset among hospitalized severe acute respiratory infection (SARI) patients and influenza like illness (ILI) outpatients, China, Febuary 1, 2011 to October 27, 2013.** Panel **A**. Four Northern Hospitals. Panel** B**. Six Southern Hospitals. *positive rate (%): (1) ILI flu positive rate = percent of influenza positive specimens among all tested specimens for influenza like illness patients. (2) SARI flu positive rate=percent of influenza positive specimens among all tested specimens for severe acute respiratory infection patients.

In the 4 hospitals in the north, the influenza activity among ILI patients peaked in the winter-spring each year, similar to influenza activity among SARI patients [Figure 5]. Also, as seen with SARI patients, the predominant types/subtypes of virus among ILI patients varied from season to season [Figure 4]. During the study period, the types/subtypes of influenza virus among ILI patients in the north were predominantly pH1N1 and B during 2011 spring months (accounting for 14% and 78% of isolates collected March-May), B during 2011–2012 winter months (accounting for 93% of isolates collected December-February), B and A(H3N2) during 2012 spring months (accounting for 73% and 31% of isolates collected March-May), A(H3N2) and pH1N1 during 2012–2013 winter months (accounting for 54% and 45% of isolates collected December-February), and pH1N1 during 2013 spring months (accounting for 64% of isolates collected March-May).

In the 6 hospitals in the south, the influenza activity among ILI patients peaked in both the winter-spring months and summer months each year [Figure 5]. In the south, the predominant types/subtypes of virus among ILI patients also differed from season to season [Figure 4]. During the study period, the types/subtypes of influenza viruses among ILI patients were predominantly pH1N1 and B during 2011 spring months (accounting for 33% and 62% of isolates collected March-May), B during 2011–2012 winter months (accounting for 81% of isolates collected December-February), B and A(H3N2) during 2012 spring months (accounting for 36% and 62% of isolates collected March-May), A(H3N2) during 2012 summer months (accounting for 97% of isolates collected July-August), pH1N1 during 2012–2013 winter months and 2013 spring months (accounting for 81% of isolates collected December-February and 86% of isolates collected March-May), and A(H3N2) during 2013 summer months (accounting for 77% of isolates collected July-August).

In both the north and the south, the predominant types/subtypes of influenza strains among SARI patients in each season were among the predominant types/subtypes of influenza strains circulating among ILI patients for that season [Figures 4 and 5].

## Discussion and conclusions

This paper describes data from the first SARI sentinel surveillance system among hospitalized patients in China, focusing on those with laboratory-confirmed influenza from February 2011 to October 2013. More than 60% of SARI patients with confirmed influenza were children < 5 years of age and adults 65 years of age and older. This is consistent with findings from other studies that demonstrate the heaviest burden of severe influenza disease within these two age groups [15,16].

Many studies have reported that seasonal influenza causes more severe disease and more often leads to hospitalization in certain populations, including children aged < 5 years, adults aged ≥65 years, pregnant women, and persons with chronic medical conditions [15,16]. In our study, a greater proportion of SARI patients with confirmed influenza had at least one underlying chronic medical condition, compared with SARI patients without influenza. Although our study did not measure risk for severe disease among all patients with influenza, the high burden of chronic disease among hospitalized SARI patients with influenza in our surveillance system is consistent with other studies. One study in the US reported 49% of children hospitalized with community-acquired laboratory-confirmed influenza had at least one chronic medical condition [17]. Another study in China reported that the prevalence of chronic medical conditions among patients with severe illness due to the 2009 pandemic H1N1 influenza infection was 33%, compared with just 14% for those with mild illness [18]*.* Having an underlying chronic medical condition also increased the risk of influenza-related death [15,16].

Our study, similar to prior studies, found that respiratory disease and cardiovascular disease were two of the most common chronic medical conditions among SARI patients with influenza [15,16,18,19]. The most common respiratory conditions in our study included chronic obstructive pulmonary disease (COPD) and chronic bronchitis. In contrast to a prior study of SARI patients in central China [20], SARI patients with confirmed influenza in our study were less likely to have asthma than patients without influenza. This finding warrants further investigation within a larger study population, however, as the number with diagnosed asthma in both SARI patients with and without influenza was low.

In our study, vaccination with seasonal influenza vaccine in the prior year was low (<3%) in both SARI patients with and without confirmed influenza. Currently, although Chinese Center for Disease Control and Prevention recommends seasonal influenza vaccination for those at high risk for severe illness from influenza, including pregnant women, young children, the elderly and persons with chronic illness [21], seasonal influenza vaccination is not included in the national immunization program. As China expands seasonal influenza vaccination in the future, data from the SARI surveillance system can inform seasonal vaccination timing decisions.

In this study, influenza activity among SARI cases in northern China increased in the colder winter months (December-March), and spring months (March-May), consistent with the distinct winter-spring peak in the north seen in other studies [4,5,19]. These data suggest that the optimal time to launch annual influenza vaccination campaigns in northern China is the fall, prior to increased influenza activity. This is consistent with the current practice in China, where influenza vaccination promotion begins in October [21]. In southern China, influenza circulated year-round, suggesting that additional studies are needed to inform optimal timing of vaccination campaigns in the south. China’s complex climate and geography and diverse socio-economic and demographic characteristics lead to varied seasonal influenza patterns, especially in southern China [6]. Continuing, improving and expanding surveillance over several years will increase understanding of influenza seasonality by region.

Data from China’s SARI surveillance system provide important evidence on types/subtypes of virus causing severe influenza illness. Though the timing of northern China’s peak influenza activity is similar to that seen in North America and Europe, the types/subtypes of predominant strains based on isolates from SARI patients in our study were not always consistent with the predominant types/subtypes identified in these other regions during the same winter influenza peaks [22-24]. Within China, however, the predominant types/subtypes of virus from SARI patients in each winter-spring influenza season were the same in the north and the south.

The predominant types/subtypes of virus causing severe influenza disease varied by year for each of the three winter peaks of this study. In the spring months, the predominant types/subtypes were the same in 2011 and 2013, but different in 2012. This temporal variation of predominant type/subtype was also seen within the ILI surveillance system [25]. Of note, the ILI surveillance system detected more than one predominant type/subtype in certain influenza seasons. The predominant type/subtype identified by the SARI surveillance system, however, was almost always among the predominant type/subtype identified by the ILI system. This finding has reassuring implications for vaccine candidate strain selection, as recommendations for strain inclusion within the vaccine are based on the ILI surveillance system.

Our study is prone to a number of limitations. First, we analyzed data from 33-months, a period not enough to predict the seasonal trends of influenza activity and to assess influenza disease burden. Second, given China’s geographic and economic diversity, data from ten sentinel hospitals are not representative of the entire country. Representativeness will improve as sentinel sites are added to this new surveillance system [26]. Third, this paper describes findings from the initial months of the SARI surveillance system in China, when data quality was inconsistent. It is likely that some cases were not captured by the system, and 7% of the CRFs were missing. Further, only 29% of SARI cases were swabbed within 24 hours of hospital admission (31% among influenza-positive SARI patients and 29% among influenza-negative SARI patients, p = 0.509) which may have increased the false negative rate for influenza. Finally, because we were not able to define the catchment populations for the ten sentinel hospitals, we were unable to calculate hospitalization rates for influenza infection. Additional health utilization surveys are necessary to assess disease burden in the future [27].

This is the first study to describe laboratory-confirmed influenza activity among SARI patients hospitalized in ten sentinel sites of China. These surveillance data inform influenza prevention and control strategies. Vaccine strain selection is based upon ILI surveillance. Reassuringly, the types/subtypes of circulating influenza strains and epidemic trends among SARI influenza inpatient cases were similar to those among milder ILI outpatient cases. Continued and expanded SARI surveillance is necessary to fully assess the disease burden of influenza, and to describe the seasonality patterns and influenza strains circulating by region.

## Abbreviations

SARI: Severe acute respiratory infection
ILI: Influenza like illness
CRF: Case report form
NP: Nasopharyngeal
VTM: Viral transport medium
CDC: Center for Disease Control and Prevention
rRT-PCR: Real-time reverse transcription PCR
pH1N1: A(H1N1)pdm09 influenza
IQR: Interquartile ranges
ICU: Intensive care unit
